# CT-based radiomics model to predict platinum sensitivity in epithelial ovarian carcinoma: a multicentre study

**DOI:** 10.1186/s40644-025-00906-9

**Published:** 2025-07-03

**Authors:** Mengge He, Rahul Singh, Mandi Wang, Grace Ho, Esther M. F. Wong, Keith W. H. Chiu, Anthony K. T. Leung, Ka Yu Tse, Philip P. C. Ip, Andy Hwang, Lujun Han, Elaine Y. P. Lee

**Affiliations:** 1 Department of Diagnostic Radiology, The University of Hong Kong, Hong Kong, China; 2 Department of Radiology, Queen Mary Hospital, Hong Kong, China; 3 Department of Radiology, Pamela Youde Nethersole Eastern Hospital, Hong Kong, China; 4 Department of Radiology & Imaging, Queen Elizabeth Hospital, Hong Kong, China; 5 Department of Clinical Oncology, Queen Elizabeth Hospital, Hong Kong, China; 6 Department of Obstetrics and Gynecology, The University of Hong Kong, Hong Kong, China; 7 Department of Pathology, The University of Hong Kong, Hong Kong, China; 8 Department of Radiology, Sun Yat-Sen University Cancer Centre, Guangzhou, China

**Keywords:** Epithelial ovarian carcinoma, Computed tomography, Radiomics, Chemotherapy, Platinum-Resistance

## Abstract

**Objective:**

Platinum resistance carries poor prognosis in epithelial ovarian carcinoma (EOC). This study aimed to assess the value of radiomics model based on contrast-enhanced CT (ceCT) in predicting response to platinum-based chemotherapy in EOC.

**Materials and methods:**

Patients with histologically confirmed EOC and pre-treatment ceCT were retrospectively recruited from 5 centres. All patients underwent standard platinum-based chemotherapy and optimal cytoreduction. Platinum sensitivity was determined by whether it recurred within six months after platinum-based chemotherapy. The whole tumour volume was manually segmented on the baseline ceCT. Radiomics features were extracted using the open-source package PyRadiomics (version 3.0.1). Patients from centres A-C were randomly divided into training and internal validation sets in 4:1 ratio. Patients from the centres D and E were assigned as independent external validation sets. Spearman’s rank correlation followed by 5-fold stratified cross validation (SCV) elastic net repeated for 100 times, and Mann-Whitney U test were deployed for feature reduction and selection. Adaptive synthetic sampling was applied to minimize class biases. Extra Trees classifier across 10-fold SCV was used for model building. The area under curve (AUC), calibration curve assessment, and decision curve analysis (DCA) were deployed to evaluate model performance and translational clinical utility.

**Results:**

Seven hundred and three EOC patients (51.6 ± 9.3 years) were recruited. The training data (*n* = 608) yielded the following classification metrics: AUC (0.917), sensitivity (83.9%), specificity (94.4%), and accuracy (91.7%) in the internal validation set. The external validation set using centre D (*n* = 44) had AUC (0.877), sensitivity (76.5%), specificity (92.6%), and accuracy (86.4%); while centre E (*n* = 51) had AUC (0.845), sensitivity (73.3%), specificity (86.1%), and accuracy (82.4%) in predicting platinum sensitivity. DCA illustrated net clinical benefit in internal validation set and both external validation sets.

**Conclusions:**

The proposed CT-based radiomics model could be useful in predicting platinum sensitivity in EOC with potential in guiding personalized treatment in EOC.

**Supplementary Information:**

The online version contains supplementary material available at 10.1186/s40644-025-00906-9.

## Introduction

The treatments for primary epithelial ovarian carcinoma (EOC) include cytoreductive surgery, platinum-based chemotherapy and targeted therapy like poly (ADP-ribose) polymerase (PARP) inhibitor and/or anti-angiogenic agents (bevacizumab) [1, 2]. Despite the initial high response rate (> 80%) after platinum-based chemotherapy, most patients will relapse with a median progression-free survival (PFS) of 18 months [3]. In the setting of platinum-sensitive recurrence, patients will usually be rechallenged by platinum-based chemotherapy with or without secondary debulking surgery, and the response rate ranged between 30 and 90% [4, 5]. Unfortunately, for patients who become platinum-refractory or platinum-resistant, the prognosis is poor with a low response rate of less than 15% to further platinum-based chemotherapy [6]. Therefore, alternative therapy like single-agent second-line chemotherapy and antibody-drug conjugates will be used [7]. Although it remains unclear of the mechanisms of platinum resistance development, the genomic diversity and the heterogeneity of the tumour microenvironment have been frequently associated with platinum resistance [7, 8]. Molecular alterations like *BRCA* mutation and homologous repair deficiency (HRD) may lead to more targeted molecular-based treatment and play vital role in determining platinum sensitivity and outcome of patients with EOC [7, 8].

Currently, there is no reliable way to predict the response to platinum-based chemotherapy prospectively until patients are being assessed after the chemotherapy. This may subject those potentially platinum-refractory and platinum-resistant patients to unnecessary platinum-based chemotherapy, resulting in chemotherapy-induced complications and delaying them in receiving alternative therapies. Therefore, a reliable and non-invasive biomarker in predicting the response to platinum-based chemotherapy before treatment initiation will help guide individualized treatment and improve the disease prognosis of EOC.

In recent years, a growing interest in exploring the association between intra-tumour heterogeneity and imaging features has emerged. Radiomics is a mathematical quantitative analysis that converts medical images into high-dimensional data by extracting large amounts of statistical features, including shape, first-order, gray level, engineered or textural features [9]. Studies have applied radiomics in tumour characterization like histological subtyping, and these radiomics features were stable to auto-segmentation [10, 11]. Previous work in EOC reported the ability of CT or MRI-based radiomics models to predict clinical outcomes and treatment response, including rate of complete tumour debulking, *BRCA* mutation status, recurrence rate and disease survival [12–17]. Yi et al. developed a combined model incorporating single-nucleotide polymorphisms with clinicopathological and CT-based radiomic features to predict platinum resistance in EOC [18]. Li et al., on the other hand, explored the value of a nomogram combining MRI radiomic features with clinical characteristics to identify platinum resistance [19].

CT is the recommended imaging modality for staging EOC [20]. At present, multicentre study for CT-based radiomics study with independent external validations is lacking. To validate the above findings based on a large cohort across multiple centres with different scanners and independent external validations will be important. Therein, the aim of this study was to assess the value of CT-based radiomic model in prediction of platinum sensitivity of EOC using multicentre data.

## Materials and methods

This multicentre retrospective study was approved by the relevant local Institutional Review Boards in accordance with the Helsinki Declaration with waived informed consent and complied with the latest Transparent Reporting of a Multivariable Prediction Model for Individual Prognosis or Diagnosis (TRIPOD) reporting guideline [21].

### Study population

Lists of patients with EOC were drawn from databases from local hospitals and collaborating institutes between January 2009 and December 2022.

**Fig. 1:**
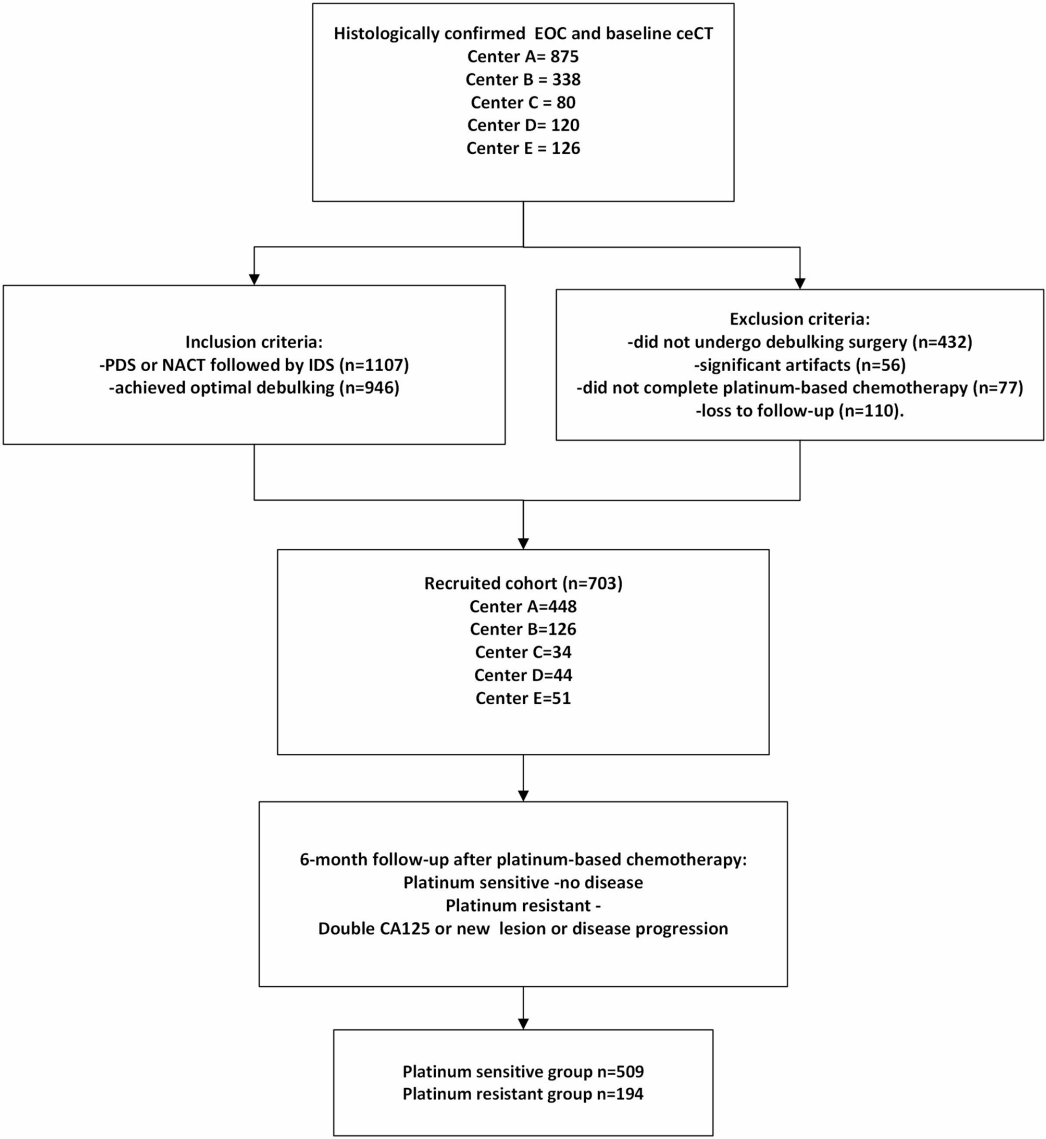
Flowchart of Patient Recruitment

The inclusion criteria included patients: (a) with histologically confirmed EOC, (b) without prior history of pelvic surgery or treatment, (c) who underwent pre-treatment contrast-enhanced CT (ceCT), (d) who underwent either primary debulking surgery (PDS) or neo-adjuvant chemotherapy followed by interval debulking surgery, (e) who achieved optimal debulking (less than 1.0 cm residual disease), (f) who received standard adjuvant platinum-based chemotherapy, (g) with follow-up of at least 6 months after completion of adjuvant platinum-based chemotherapy. The exclusion criteria were those with (a) significant artefacts on ceCT images precluding assessment of the primary tumour in the pelvis, (b) no available clinical follow-up data, (c) tumour diameter smaller than 1.0 cm (Fig. 1). Patient electronic system was retrospectively reviewed for at least 6 months after the last dose of adjuvant platinum-based chemotherapy. Disease recurrence within the first 6 months was confirmed with a rise in CA125 (double from the upper limit), newly found lesion or any residual lesion with a magnitude increase of over 20% compared with post-operative imaging. Patients were dichotomized into platinum-sensitive and platinum-resistant groups, defined as follows for the purpose of classification [8]:

- platinum-sensitive: no disease recurrence within the first 6 months of completion of platinum-based chemotherapy.
- platinum-resistant: refractory disease with disease progression while on platinum-based chemotherapy or disease recurrence within 6 months after completion of platinum-based chemotherapy.

### Imaging acquisition

Pre-treatment abdominopelvic ceCT scans were acquired on multiple scanners in 5 centres. The CT scanners and imaging parameters from centres A-D are tabulated in Table S1. The CT images from centre E were anonymized open-source data from the Cancer Imaging Archive TCIA (https://cancerimagingarchive.net) [22].

### Tumour segmentation

All the ceCT images were normalized and resampled to 5 mm slice thickness for tumour segmentation and radiomics analysis. Tumour segmentation was manually delineated on open-source platform ITK-SNAP (3.6.0, http://www.itksnap.org/pmwiki/pmwiki.php) [23].

Two radiologists who were blinded to the clinicopathological information (R1 and R2, both with 5 years’ experience in pelvic imaging) delineated the tumours on ceCT images. Both contoured around the borders of the tumour on each slice containing the tumour, encompassing both the cystic and solid components. Subsequently, all the segmentations were verified by a third radiologist (R3, > 15 years’ experience in pelvic imaging). Any disagreement in tumour segmentation was resolved in consensus. If bilateral ovarian masses were detected, only the larger ovarian mass was delineated for radiomics analysis.

### Radiomic features extraction

Training and internal validation were based on data from centres A-C with a split ratio of 4:1, while external validations were performed using data from centres D and E, respectively. Radiomics features were extracted using the open-source package PyRadiomics (version 3.0.1, https://pypi.org/project/pyradiomics/), adhering to the Image Biomarker Standardization Initiative (IBSI) guidelines [24]. All images and their corresponding masks were resampled to isotropic voxel spacing of 1 mm × 1 mm × 1 mm using B-spline interpolation on-the-fly during feature extraction. Seven classes of radiomic features were extracted from ceCT images, namely shape, first-order, gray-level co-occurrence matrix (GLCM), gray-level size zoon matrix (GLSZM), gray-level run length matrix (GLRLM), gray-level dependence matrix (GLDM) and neighbouring gray tone difference matrix (NGTDM) features. In addition, Laplacian of Gaussian (LoG) features with sigma of 1 to 5 [25], and wavelet features with transformed images that yielded 8 decompositions were also extracted. In total, 1134 radiomics features were extracted for each patient.

### Pre-processing and features reduction

Feature scaling was performed using z-score standardization followed by power transformation to approximate a Gaussian distribution. Each pair of features with a Spearman’s rank correlation coefficient (*r* > 0.9), one feature was retained, and the other was excluded to mitigate multicollinearity while minimizing the loss of potentially important information. This would reduce redundancy and potential scanner-related noise.

### Feature selection and data augmentation

Highly repeatable features were identified using elastic net regression, validated across 5 stratified folds and repeated 100 times to eliminate feature selection bias; detailed hyperparameter settings are provided in Supplementary S1. Subsequently, Mann-Whitney U test at a significance level of 0.05 was used to select statistically significant features. Adaptive Synthetic Sampling (ADASYN) was deployed for data augmentation to mitigate data biasness, as the data were heavily imbalanced towards platinum-sensitive cases.

### Model Building and evaluation

We investigated different machine learning (ML) classifiers, including traditional classifiers such as Logistic Regression (LR), Decision Tree (DT), Support Vector Machine (SVM), K-Nearest Neighbors (KNN), Gaussian Naive Bayes (GNB), Linear Discriminant Analysis (LDA), etc. and ensemble-based methods, including bagging and boosting strategies such as Extreme Gradient boosting (XGBoost), Light Gradient Boosting Machine (LightGBM), Categorical Boosting (CatBoost), Random Forest (RF), AdaBoost, Gradient Boosting and Extra Trees. Hyperparameters were optimized using Stratified 10fold GridSearchCV for each of these models. The predictive performance of these models was quantified by receiver operating characteristic (ROC) curves, with the area under the curve (AUC) and corresponding 95% confidence intervals (CI) and the best model was selected based on highest mean cross-validation AUC. The efficacy of best performing model was quantitatively evaluated using AUC, accuracy, sensitivity, specificity, precision and F1 score on internal validation cohort and 2 external validation cohorts. Delong test was conducted to compare statistical differences between the ROC curves against best model. Calibration curves were constructed to illustrate the agreement between predicted and observed probabilities and Hosmer − Lemeshow test was conducted to evaluate the goodness of-fit. The decision curve analysis (DCA) was incorporated under different threshold probabilities to evaluate the clinical utility of the model. SHAP (SHapley Additive exPlanations) analysis was performed to interpret the feature contributions within the best-performing model. This method assigned an importance value to each feature for every prediction, enabling us to systematically assess their influence. The analysis revealed which radiomic features had the greatest impact on the model’s output.

**Fig. 2:**
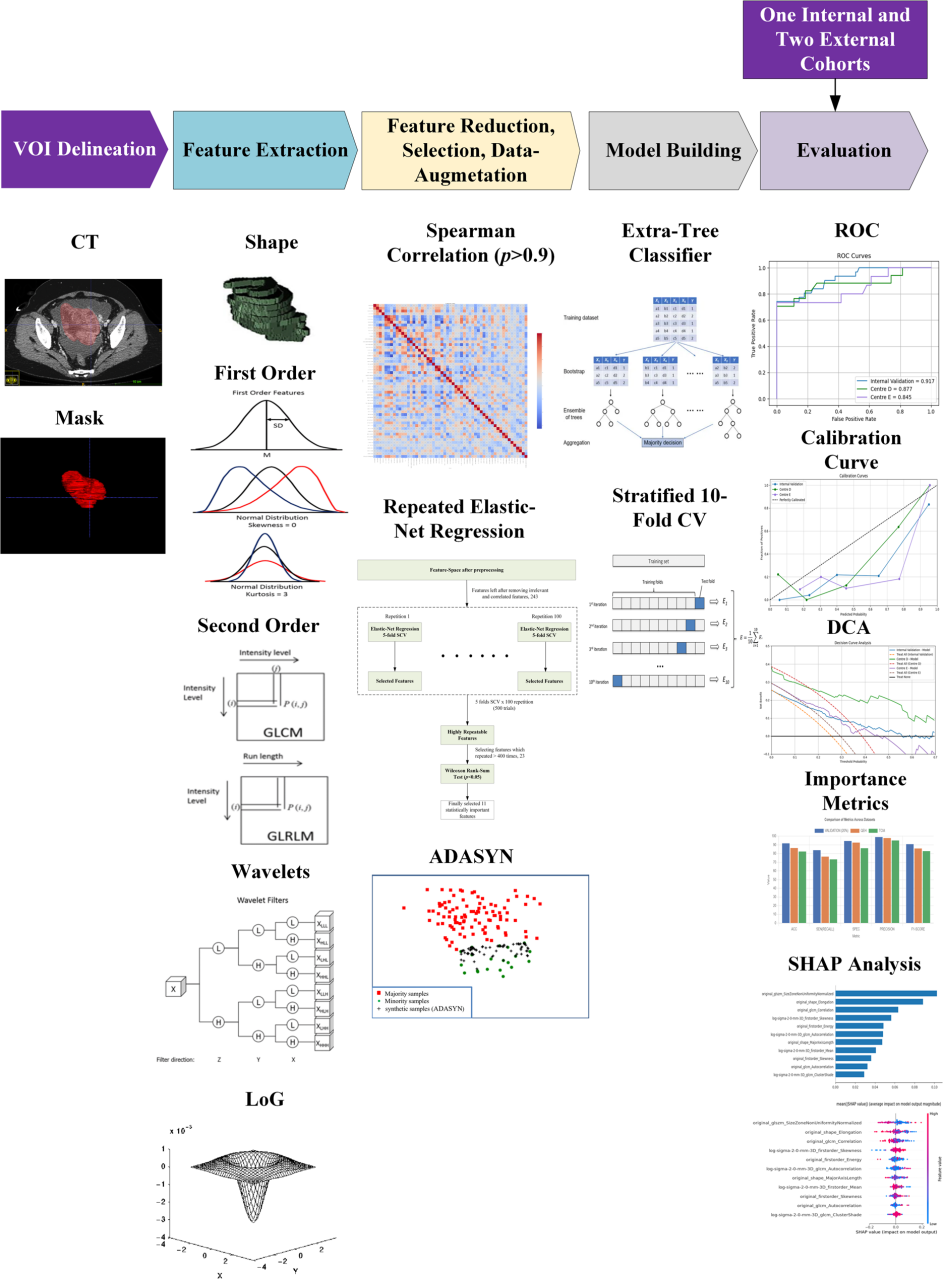
Workflow Diagram of Radiomics Model Pipelines to Predict Platinum Sensitivity in EOC

Statistical analyses were performed using the following Python libraries: scikit-learn (version 1.2.1), NumPy (version 1.23.5), imbalanced-learn (version 0.10.1), Matplotlib (version 3.7.1), and SHAP ( version 0.42.1). All analyses and model development steps were executed within a Python-based environment (Python 3.9.16). The schematic flowchart of the proposed pipeline is illustrated in Fig. 2.

## Results

### Demographics

A total of 703 patients (51.6 ± 9.3 years, range 18–90) newly diagnosed EOC patients from 5 centres were recruited between January 2009 and February 2023. The patients’ demographics and response to platinum-based chemotherapy are shown in Table 1. Among these patients, 501(71.3%) patients had serous adenocarcinoma, among them 472 (67.1%) had high-grade serous tumours (HGSOC) with a platinum-sensitive rate of 70.9%; 73(10.4%) patients had endometrioid adenocarcinoma with a platinum-sensitive rate of 84.9%; 84(11.9%) patients had clear cell carcinoma with a platinum-sensitive rate of 70.2%; 31(4.4%) patients with mucinous carcinoma had platinum-sensitive rate of 93.5%; 14(2.0%) remaining patients were of mixed subtype tumours or only known as ovarian adenocarcinoma, with a platinum-sensitive rate of 64.3%. Regarding the International Federation of Gynecology and Obstetrics (FIGO) stages, 127 (18.3%) patients were in stage I; 79 (11.4%) patients were in stage II; 341(49.1%) patients were in stage III; 147 (21.2%) patients were in stage IV; while other 10 cases were missing FIGO staging.

**Table 1:** Demographics and platinum sensitivity of patients with EOC

Demographics	Patients (*n*)	Age (mean ± sd)	Platinum-sensitive	Platinum-resistant
Total	703	53.5 ± 9.6	509(72.4%)	194(27.6%)
Centre A	448	55.8 ± 9.8	338(75.4%)	110(24.6%)
Centre B	126	50.1 ± 12.5	87(69.0%)	39(31.0%)
Centre C	34	54.3 ± 11.7	21(61.8%)	13(38.2%)
Centre D	44	52.9 ± 10.7	27(61.4%)	17(38.6%)
Centre E	51	53.6 ± 8.1	36(70.6%)	15(29.4%)
SC	501	55.5 ± 13.4	355(70.9%)	146(30.1%)
EC	73	49.9 ± 10.2	62(84.9%)	11(15.1%)
CCC	84	53.1 ± 11.8	59(70.2%)	25(29.8%)
MC	31	49.8 ± 10.7	29(93.5%)	2(6.5%)
Others	14	56.8 ± 11.5	9(64.3%)	5(35.7%)

Abbreviation: EOC, epithelial ovarian carcinoma; CCC, clear cell carcinoma; SC, serous adenocarcinoma; MC, mucinous adenocarcinoma; EC, endometrioid carcinoma; Others, mixed subtypes or only known as ovarian adenocarcinoma

### Selected radiomic features and performance of selected model

**Fig. 3:**
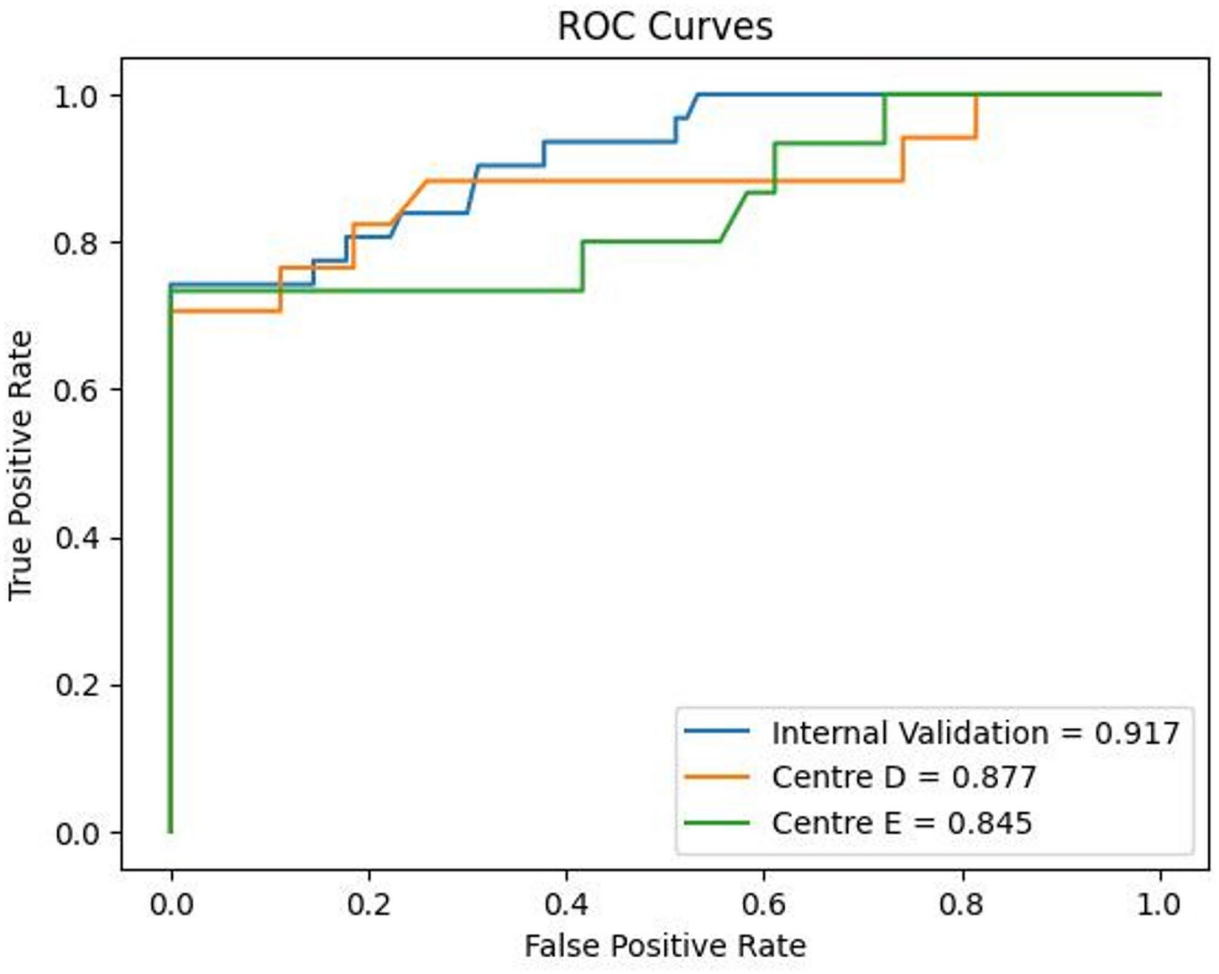
ROC Curves for Internal and the Two External Validation Cohorts of the ET model

**Fig. 4:**
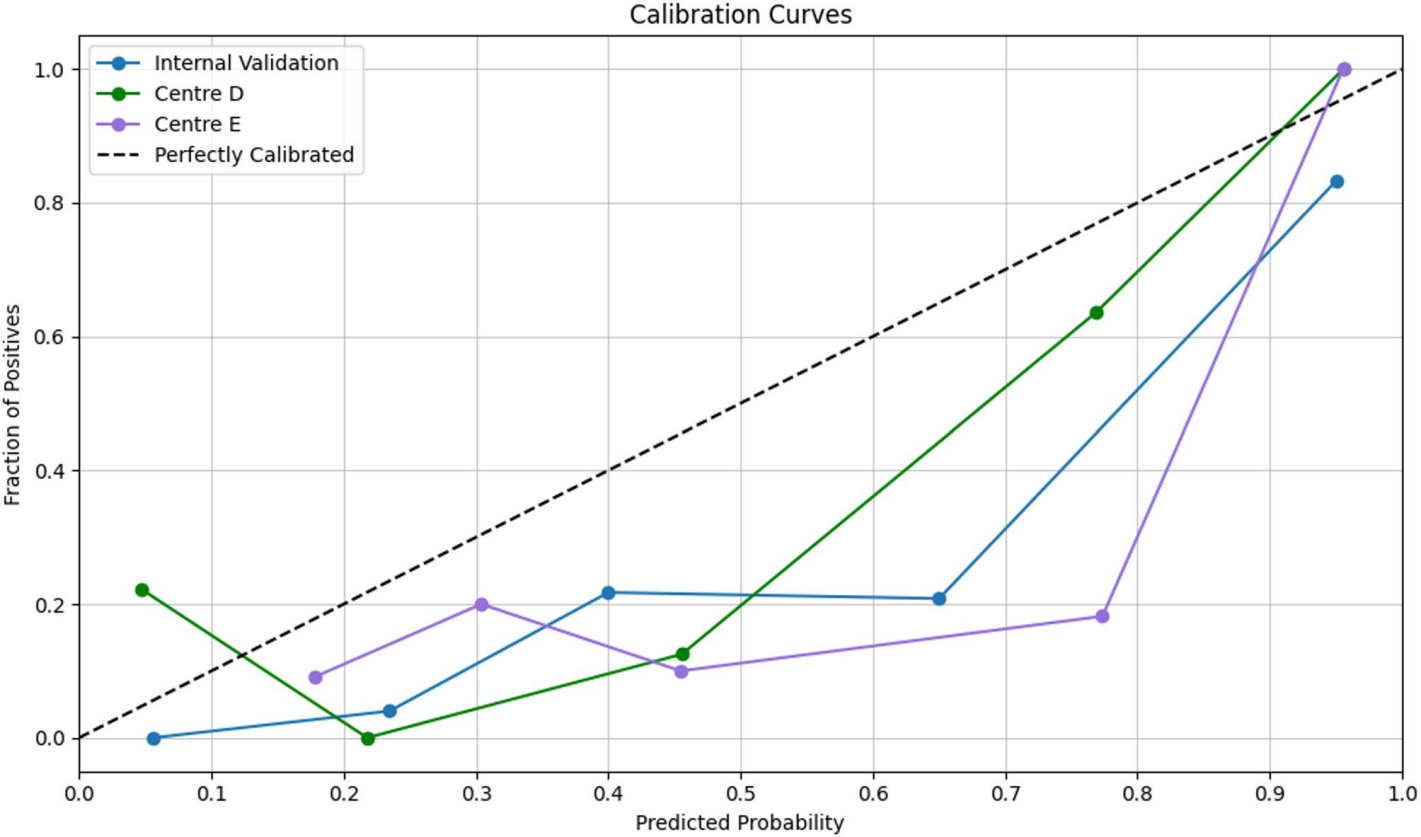
Calibration Curves for Internal and the Two External Validation Cohorts of the ET model

All classifier were built based on 11 statistically significant radiomic features with their discriminative ability in determining response to platinum-based chemotherapy. ET classifier demonstrated superior predictive performance compared with traditional ML algorithms and tree- based ensemble methods. DeLong’s test was employed to evaluate statistical differences between the ROC curves and analysis revealed that the ET classifier achieved significantly discriminative performance compared to both traditional ML classifiers and other tree-based ensemble approaches and results are reported in Table S2. The AUCs of ET classifier model for predicting response to platinum-based chemotherapy were 0.917 (95% confidence interval [CI]: 0.850–0.970) for internal validation set, 0.877 (95% CI, 0.748–0.984) and 0.845 (95% CI, 0.718–0.968) for the two external validation sets, respectively. The ROC performances including AUC, accuracy, sensitivity, specificity, F1 score and precision are summarized in Table 2. The ROC curves and calibration curves of training and testing cohorts are presented in Figs. 3 and 4 respectively.

**Table 2:** Performance of extra tree model

Dataset	AUC	Accuracy	Sensitivity	Specificity	F1 score	Precision
**Internal validation**	0.917	0.917	0.839	0.944	0.839	0.839
**Centre D**	0.877	0.864	0.765	0.926	0.813	0.867
**Centre E**	0.845	0.824	0.733	0.861	0.710	0.688

**Fig. 5:**
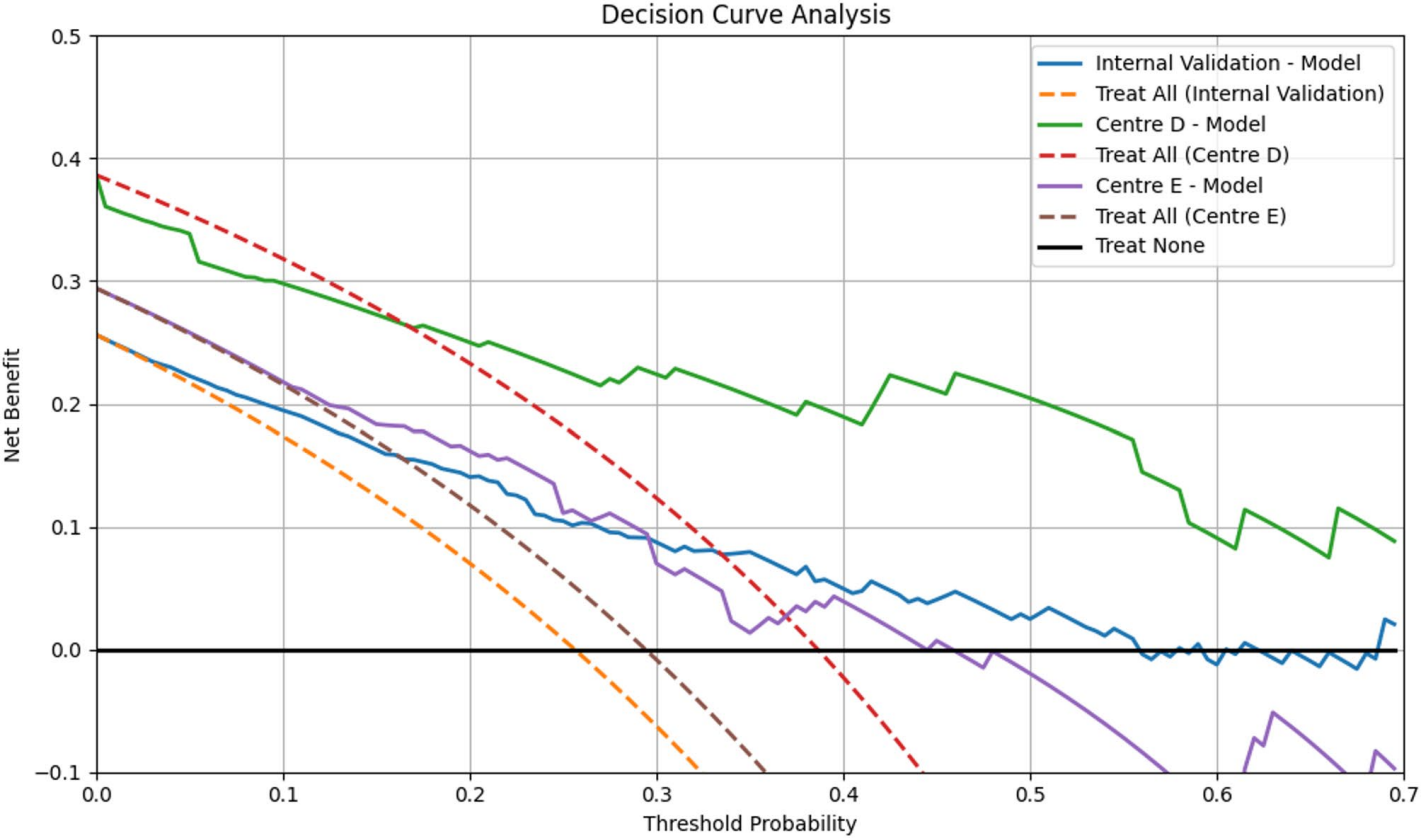
Decision Curve Analysis. Decision curve analysis for the proposed ET model in predicting platinum sensitivity in EOC

We assessed calibration quantitatively using the Hosmer–Lemeshow goodnessoffit test on the internal validation cohort and both external cohorts (Centre D and Centre E); in each case, pvalues exceeded 0.05, indicating no evidence of miscalibration and confirming consistent model fit across datasets. The threshold to evaluate the net clinical benefit for DCA was based on the prevalence of platinum-resistant ovarian carcinoma being estimated around 25% [26] and the DCA plots showed net benefits of the proposed model. Additionally, it demonstrated net benefit over ‘treat all’/‘treat none’ strategies at threshold probabilities of 0.17–0.43. This range can be defined as ‘high risk,’ to account for clinical variability in risk tolerance as illustrated in Fig. 5.

**Fig. 6:**
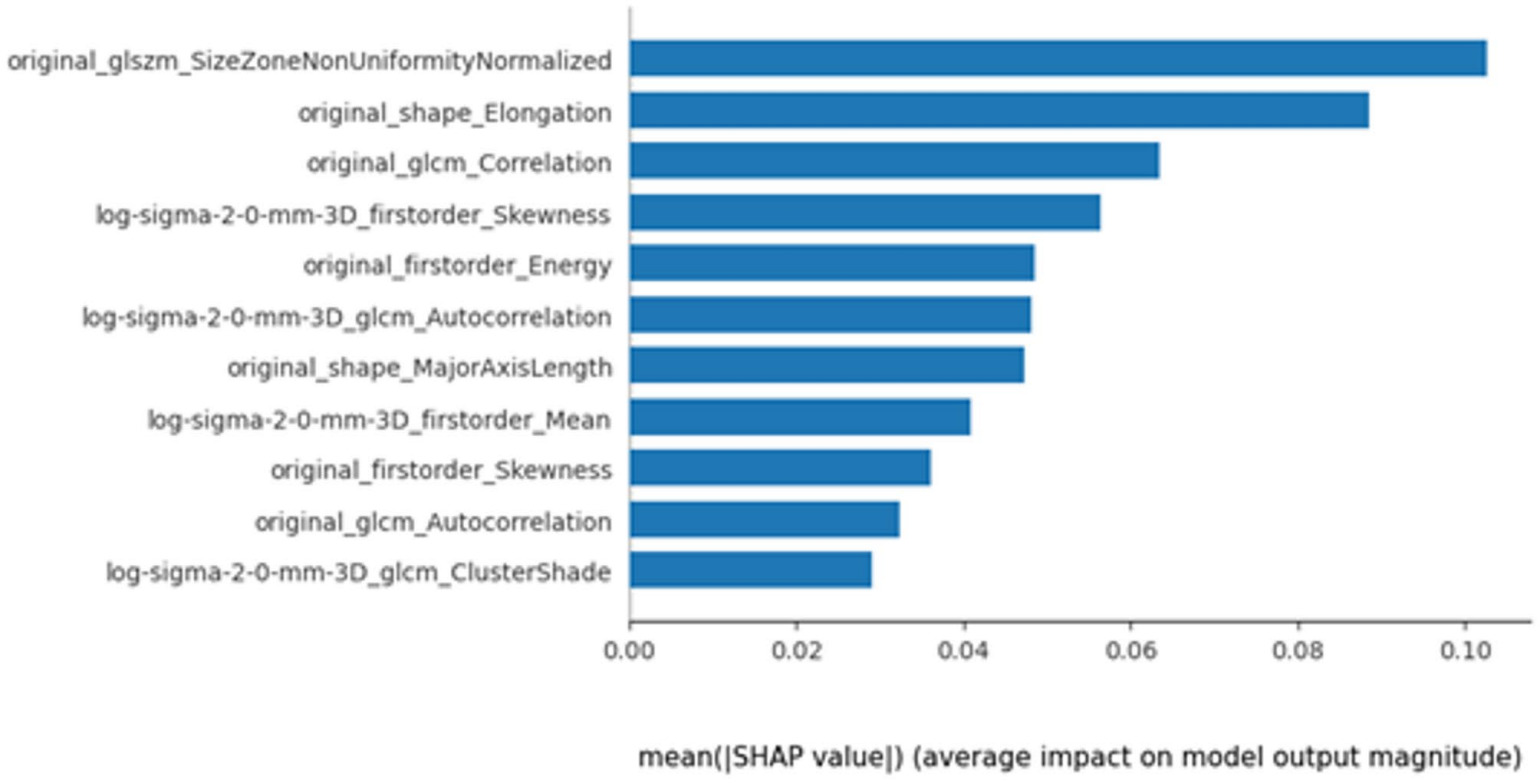
Global Measure of Feature Importance based on mean absolute SHAP values

SHAP analysis was performed to enhance model explainability and quantify the importance of radiomic features, The model’s coefficients inherently described global feature importance, whereas SHAP quantified individual feature contributions to single predictions and confirmed the consistency of the global feature ranking. The importance of each feature based on mean magnitude SHAP values in distinguishing platinum-sensitive and resistant is illustrated in Fig. 6.

**Fig. 7:**
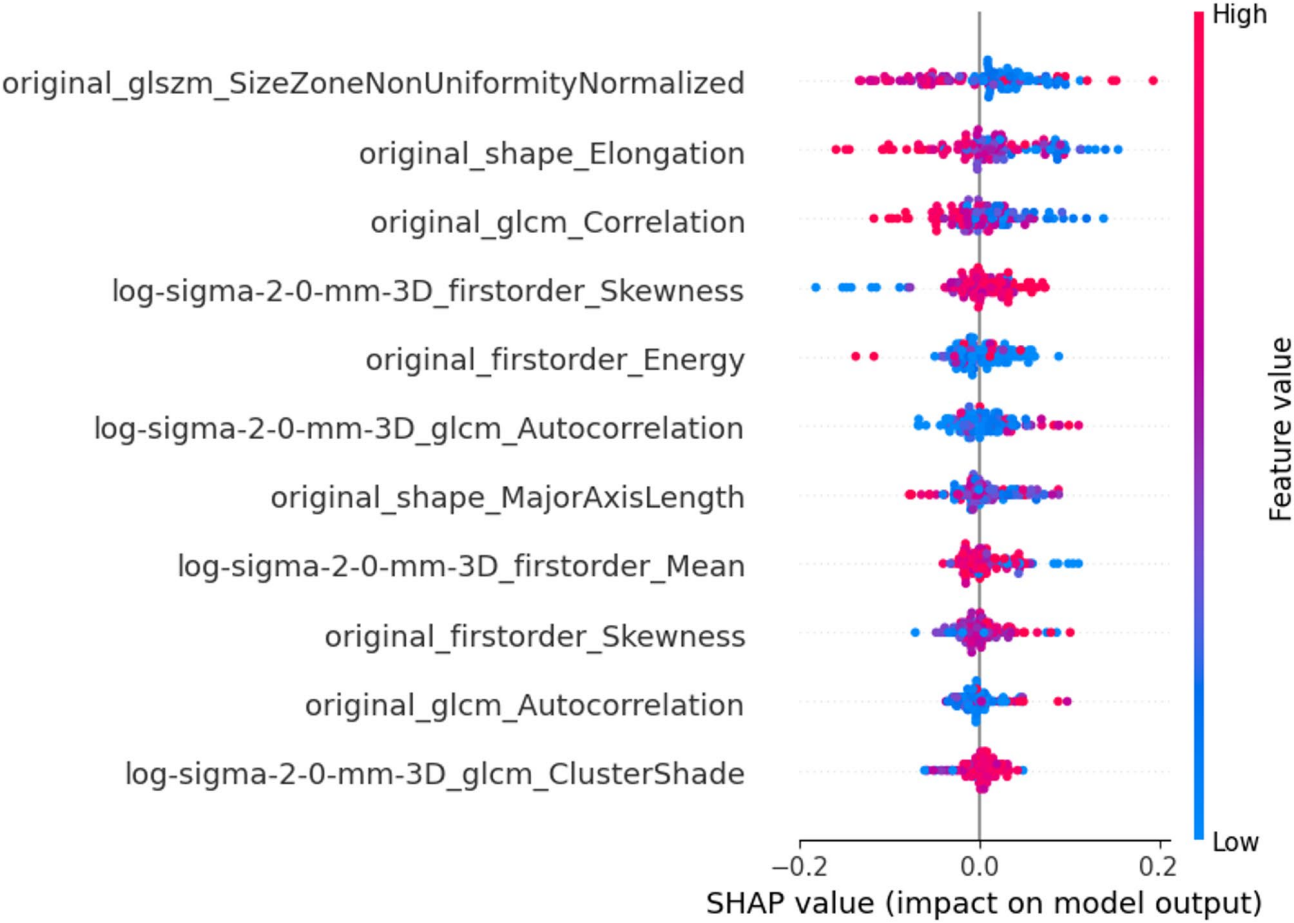
The Beeswarm Plot of Selected Features. Each point in the beeswarm plot represents the SHAP value for each patient in the internal and external centres corresponding to a specific feature. The color gradient reflects the feature value, while the horizontal position indicates the impact on the prediction—positive SHAP values push the prediction toward platinum resistant, and negative values toward platinum sensitive as evident from below figures

Additionally, the range of feature values seen for each patient for all features is illustrated using Bee-swarm plot (Fig. 7). The impact of feature importance for all patients and individual patient from each group are illustrated in Fig. S1 and Fig. S2 respectively. The clinical association of frequently identified features in platinum sensitive and resistant subgroups is also discussed in Supplementary 3.

## Discussion

This multicentre study constructed a CT-based radiomics model with excellent performance in predicting response to platinum-based chemotherapy in patients with EOC and validated its good discriminative ability in two independent external test cohorts. Notably, the two independent external test datasets were from a local hospital and an overseas open-source dataset, suggesting the generalizability of the model in a wider population.

Platinum-based chemotherapy is the mainstay chemotherapy regimen for EOC. Despite good response initially, it is inevitable that patients will acquire resistance to platinum-based chemotherapy through a complex multifactorial process. Being a heterogenous entity, less common histologies of EOC, especially mucinous and clear cell carcinoma, are less responsive to platinum-based chemotherapy [27]. Various biomarkers for platinum-resistance have been reported but results were inconsistent [28]. Baseline serum CA125 levels and FIGO staging were reported to be associated with platinum sensitivity especially in HGSOC [19]. Further studies showed that laterality of ovarian lesions [29] and FIGO stage [30] were predictive of the response to platinum-based chemotherapy. In contrary, Lu et al. found that Ki-67, rather than age, FIGO, residual lesions, laterality of ovarian lesions and CA125 levels, was useful in predicting platinum sensitivity [31]. Immunohistochemistry-derived markers such as Glypican-3, ALDH1A1, TNFR2, STAT3, FOXP3 and TIM3 were increasingly reported as promising biomarkers to predict platinum resistance in EOC patients [32]. However, these are dependent on tumour tissues availability, usually through invasive procedures, and the results suffer from variability of the respective analyses.

Radiomics, a high-throughput mining of quantitative image features from medical imaging, could act as non-invasive biomarker to improve diagnostic pathway, disease prognostication and treatment outcome prediction [33]. In the current study, 11 radiomic features from 4 classes of shape, first-order, GLCM and GLSZM features were selected for modelling, including both original and transformed features. Among these, the original Size-Zone Non-Uniformity Normalized (SZNN), being the top feature selected, measures the variability of size zone volumes throughout the image. Its higher value quantifies texture heterogeneity and might be associated with intra-tumour heterogeneity based on frequency distribution and spatial complexity of the gray-level [34]. It demonstrated higher SHAP values in the platinum-resistant group, suggesting more heterogeneous tumor texture. This would be concordant to the observations that higher intratumoral heterogeneity was linked to treatment resistance [35]. Original shape elongation can be associated with elongated tumor shapes and often correlate with invasive growth patterns and higher metastatic potential, and in our study, it was linked to platinum resistance [35, 36]. Higher energy may be associated with heterogenous legions. Study on hepatocellular carcinoma may have implicitly linked histogram energy to a biological context where high-intensity foci (e.g. necrosis, hyper-enhancement) increased Energy in heterogeneous lesions [37]. Both original skewness and transformed LoG skewness with the sigma of 2 appeared frequently in the current prediction model. A high value of skewness reveals a more asymmetric distribution, which may be related to tumour heterogeneity. Higher skewness may indicate differences in the tissue components, e.g. necrotic regions, which may altered drug penetration and treatment response due to irregular cellular distribution. Such patterns have been linked to platinum resistance in epithelial ovarian cancer. Our finding concurred with Lu et al., which found skewness derived from MRI-derived apparent diffusion coefficient map as an independent risk factor for platinum resistance with a prediction AUC of 0.733 [31]. Higher cluster shade in the log-filtered (σ = 2.0 mm) GLCM denotes more complex, asymmetric texture higher cluster shade indicates more complex, asymmetric texture. In the text we connect this to known biology: for instance, texture asymmetry may reflect underlying heterogeneity [35, 38]. However, Low gray-level co-occurrence matrix (GLCM) correlation is associated with aggressive tumours and many of the HGSOC being more sensitive to platinum-based chemotherapy is notorious being heterogenous and aggressive at presentation. Nevertheless, this seems to contradict the findings above, therefore more work is required to elucidate the biophysical meaning of these features. Moreover, radiomic features capturing tumour heterogeneity were associated with certain biomarkers and proteins in HGSOC [39, 40]. In our predictive model, many of the selected radiomic features dealing with tumour heterogeneity could potentially represent underlying tumour biology.

A “radiomic prognostic vector” comprising 4 CT radiomic features was significantly associated with primary platinum resistance, shorter PFS, and poor surgical outcome in HGSOC [41]. An integrated model combing single-nucleotide polymorphisms, clinicopathological and CT radiomic features improved prediction of platinum resistance in EOC when compared to radiomics model alone but lack independent external validation to confirm the findings [18]. A separate study devised a radiomics nomogram model, combining MRI-based radiomic features with clinical characteristics (FIGO stage, CA-125, and residual tumour), outperformed the clinical model alone (AUC: 0.799 vs. 0.747) [19]. By dividing the whole tumour volume into multiple sub-regions using K-means clustering to generate the habitat radiomics model, Bi et al. reported a higher AUC (0.710) compared to the classic radiomics model (0.640) and deep learning model (0.603) [29]. Higher prediction AUC was achieved when peritumour radiomic signatures were included compared to tumour radiomic features alone (0.720 vs. 0.671) in predicting platinum sensitivity [30]. Results from this study adds to the current literature with the advantage of the use of multicentre data and independent external validations to ensure generalizability of the proposed model.

Despite promising value of radiomics analysis in predicting the treatment response to platinum-based chemotherapy in EOC as aforementioned, several challenges and translational gap between ground truth and clinical applications should be considered, especially with result reproducibility and transferability [42, 43]. Standardization of the radiomics analysis pipeline, data sharing and collaborative research from multi-institutions will be useful approaches to facilitate radiomics adoption into clinical practice [44]. Besides, clinical integration of the proposed model would require automation and real-time processing. Therefore, auto-segmentation and auto-processing of feature extraction and model building would hold potential in handling a massive amount of data with self-leant capabilities [11].

### Limitations

This study had a few limitations. First, we did not test the intra-observer or inter-observer variability in tumour segmentation in this study, but this had been previously shown to be high with the same observers [10]. An automated segmentation tool could further improve the efficiency and stability of quantitative imaging analysis [11]. Second, clinicopathological markers such as CA125 and Ki 67 were not incorporated in the model building, as these data were inconsistently available across different centres being a retrospective study. Third, this study lacked long-term prognostic value as the long-term follow-up data was missing in many of these patients across different centres, who were being subsequently followed-up by local hospitals elsewhere after the initial surgery and completion of the chemotherapy. Fourth, we recognize that some of the radiomic features lack biological interpretability. Nevertheless, there is increasing evidence linking imaging data to biology in EOC to enhance our understanding of these radiomic features [39, 40]. Finally, this was a retrospective multicentre study, which would limit the level of evidence. A well-designed prospective study will improve the applicability of radiomics as a discipline in the area.

## Conclusions

The proposed CT radiomics model could be useful in predicting response to platinum-based chemotherapy in EOC with the potential in guiding personalized treatment in EOC. CT radiomics could act as potential biomarker of determining platinum-sensitivity in EOC.

## Electronic supplementary material

Below is the link to the electronic supplementary material.

Supplementary Material 1

## Data Availability

No datasets were generated or analysed during the current study.

## Abbreviations

EOC: Epithelial ovarian carcinoma
ceCT: Contrast-enhanced CT
PARP: Poly (ADP-ribose) polymerase
PFS: Progression-free survival
HRD: Homologous repair deficiency
TRIPOD: Transparent Reporting of a Multivariable Prediction Model for Individual Prognosis or Diagnosis
PDS: Primary debulking surgery
FIGO: International Federation of Gynecology and Obstetrics
HGSOC: High-grade serous tumours
GLCM: Gray-level co-occurrence matrix
GLSZM: Gray-level size zoon matrix
GLRLM: Gray-level run length matrix
GLDM: Gray-level dependence matrix
NGTDM: Neighbouring gray tone difference matrix
LoG: Laplacian of Gaussian
SZNN: Non-Uniformity Normalized
AI: Artificial intelligence
