# Supplementary material for: CT-based radiomics model to predict platinum sensitivity in epithelial ovarian carcinoma: a multicentre study
Source: Cancer Imaging. 2025 Jul 3;25:85. doi: 10.1186/s40644-025-00906-9 (PMC12225207; doi:10.1186/s40644-025-00906-9)
Supplement: Supplementary file 1 — Supplementary Material 1 [file 40644_2025_906_MOESM1_ESM.docx]

**Supplementary Materials**

**SECTIONS**

**Supplementary 1. Feature Selection**

The hyperparameters for elastic net regression, specifically **α** (the regularization strength) and the **L1** ratio (which controls the balance between Lasso and Ridge regularization), were determined through a rigorous grid search within a 5-fold SCV. This process was repeated 100 times to account for variability in feature selection, ensuring that the chosen hyperparameters were robust against overfitting and consistently minimized validation error.

- **α**: This parameter controls the overall strength of regularization. A higher α means stronger regularization, which can lead to more coefficients being set to zero (in the case of Lasso) or reduced (in the case of Ridge). In our study, optimizing αα was crucial to balance model complexity and predictive power.
- **L1 Ratio**: This parameter determines the proportion of L1 regularization (Lasso) to L2 regularization (Ridge). An L1 ratio of 1 corresponds to pure Lasso, while an L1 ratio of 0 corresponds to pure Ridge. By adjusting this ratio, we can control the degree of sparsity versus shrinkage in the model. For datasets with highly correlated features, a lower L1 ratio (more Ridge-like behavior) helps to reduce multicollinearity issues.

To determine the optimal values of **α** and **L1 ratio**, we conducted a grid search within a 5-fold stratified cross-validation framework, repeated 100 times to ensure robustness. The grid included:

' **α** ': [0.01, 0.1, 0.2, 0.3, 0.4, 0.5, 0.6, 0.7, 0.8, 0.9, 1.0,2,3,4 ,5,7, 10]

'**L1_ratio'**: [ 0.1, 0.2, 0.3, 0.4, 0.5, 0.6, 0.7, 0.8, 0.9, 1.0, 1.5, 2,2.5, 3.5, 4,4.5, 5]}

The grid search identified **α = 0.01** and **L1 ratio = 0.7** as the optimal hyperparameters, consistently minimizing validation error.

This process identified the combination of alpha and L1 ratio that minimized validation error, ensuring stable feature selection.

**Supplementary 2. Model Building**

To determine the optimal hyperparameters for Extra Trees Classifier, we employed a grid search approach. The hyperparameter space was exhaustively explored using the following ranges:

- n_estimators: Tested values ranged from 25 to 500 in increments of 25.
- criterion: Both 'gini' and 'entropy' were evaluated.
- max_depth: Values from 5 to 25 were considered, along with None to allow for full tree growth.

**Supplementary 3. SHAP (SHapley Additive exPlanations) Analysis**

The mean SHAP values for all patients in each group were illustrated in **Figure S1**. The mean SHAP value reflected the average contribution of each selected radiomic feature to the model’s predictions across all patients within a group. The influence of features on the model’s predictions for representative patient from each group was further demonstrated in **Figure S2** (water-fall plot).

It is evident from **Figure S1(a)** and **Figure S2(a)** that negative SHAP values for features such as original_shape_Elongation, original_glcm_Correlation, original_glcm_Autocorrelation, original_firstorder_Energy, and original_shape_MajorAxisLength consistently contributed to the model’s prediction of platinum sensitivity. These negative SHAP values indicated that lower values of these features pushed the prediction towards the platinum sensitive.

Conversely, **Figure S1(b)** and **Figure S2(b)** showed that positive SHAP values for features including log-sigma-2-0-mm-3D_firstorder_Skewness, original_glszm_SizeZoneNonUniformityNormalized, original_shape_MajorAxisLength, original_firstorder_Energy, and original_shape_Elongation led the model to predict platinum resistance. The positive SHAP values demonstrated that higher values of these features increased the likelihood of a platinum resistant prediction.

**FIGURES**

| *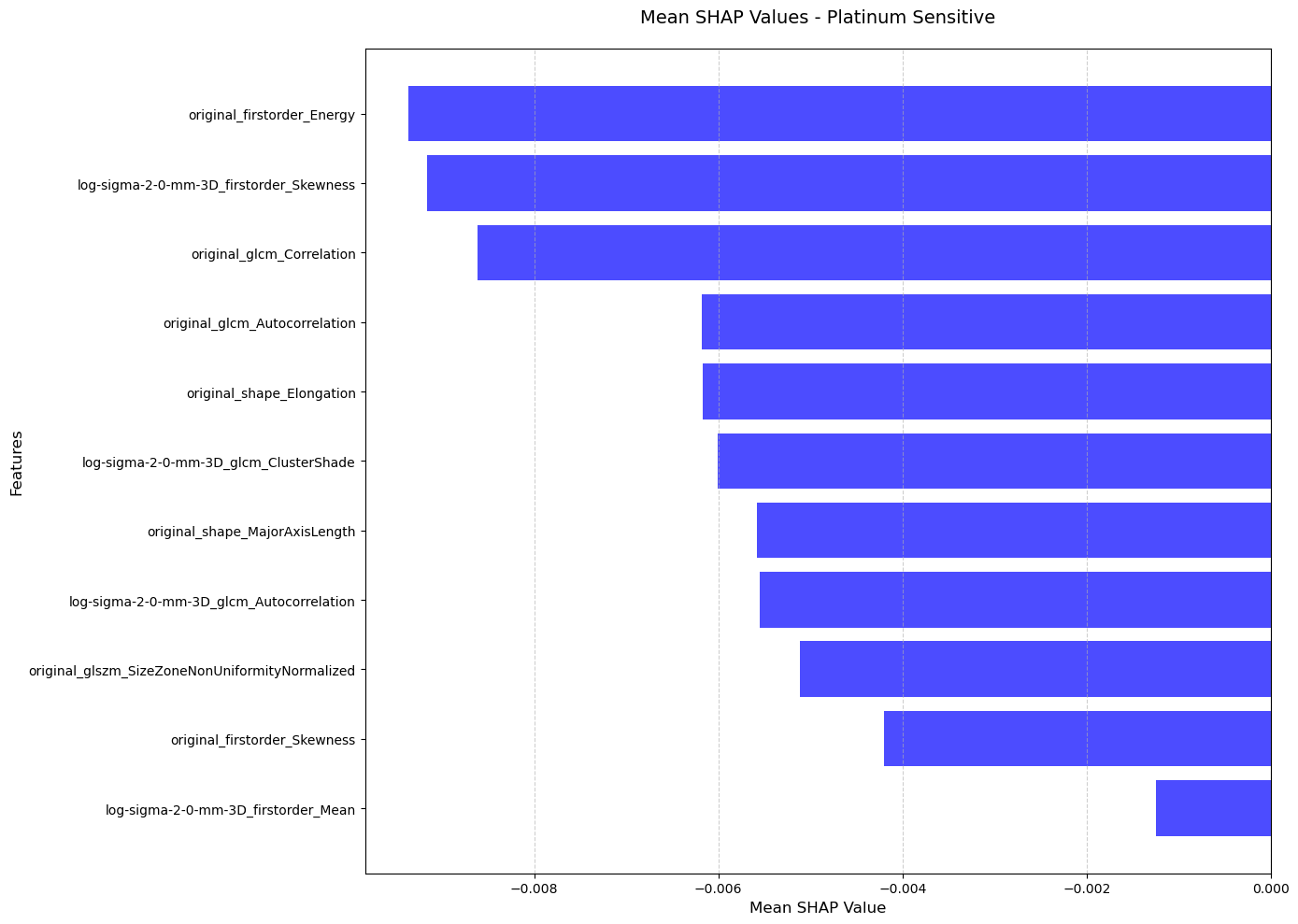* | *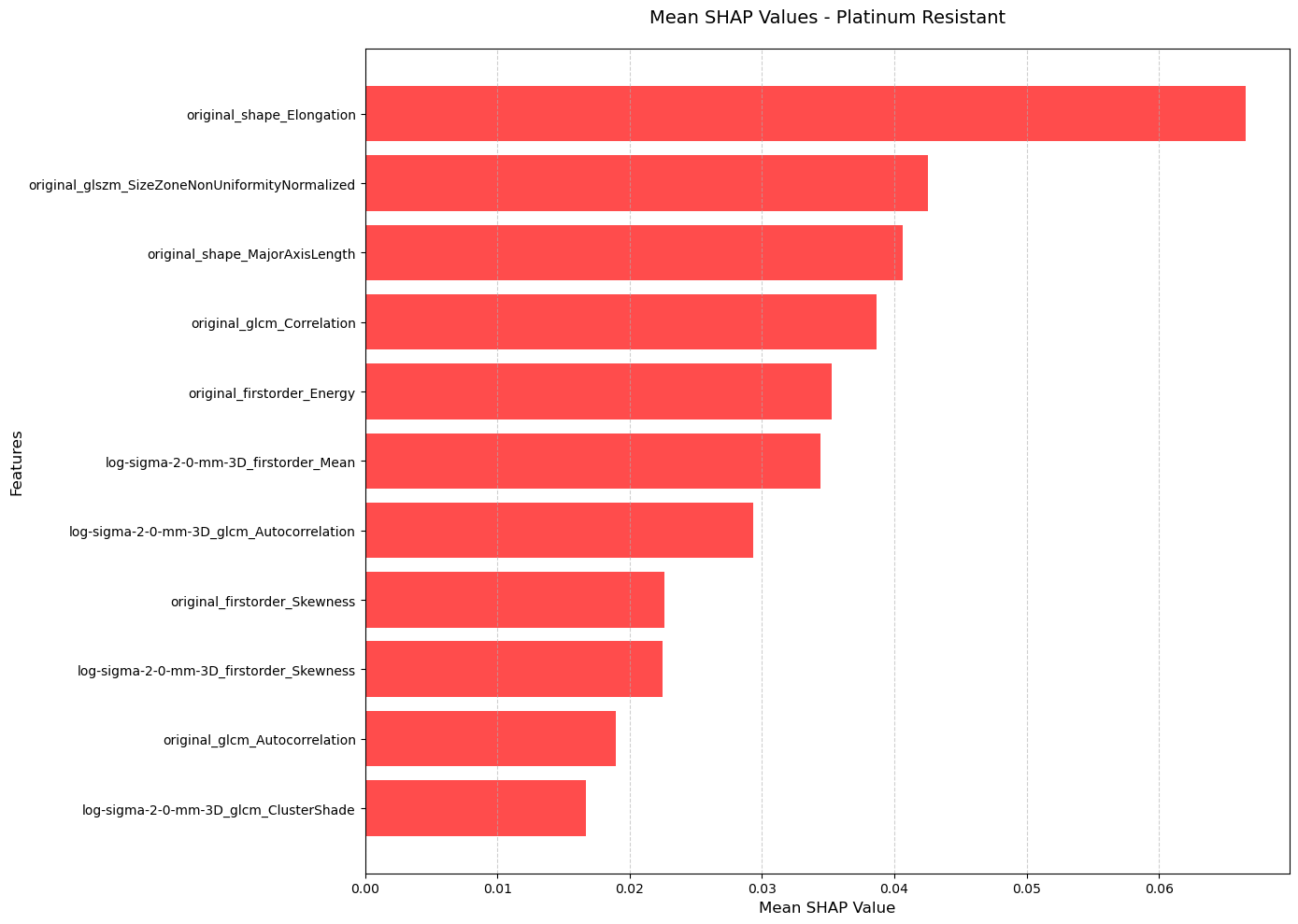* |
| --- | --- |
| 1. *platinum sensitive* | *(b) platinum resistant* |
| *Figure S1. Contribution of Mean Shap value of each feature for each group^[[1]](#footnote-1)^* | |

| *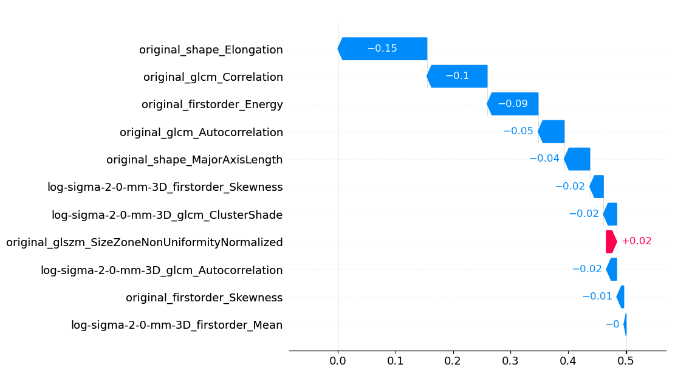* | *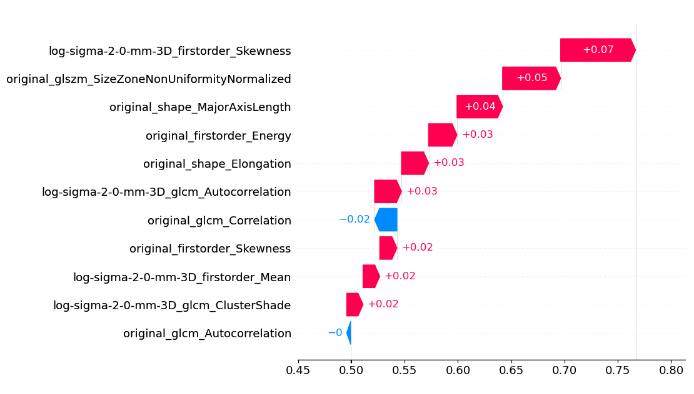* |
| --- | --- |
| (a) *platinum sensitive* | *(b) platinum resistant* |
| *Figure S2. Water fall plot for representative patient from each group* | |

**TABLES**

The CT scanners and imaging parameters from centres A-D are tabulated in **Table S1**. We have demonstrated quantitative comparative analysis for top five tree-based ensemble classifiers and traditional machine learning (ML) across the three test datasets (internal validation, Centre D, and Centre E).

The 95% confidence intervals for the AUCs of all models along the DeLong test on the ROC curves to statistically compare the AUCs between ET and other considered models are reported in **Table S2**. This analysis demonstrated ET achieved significantly higher AUCs than both tree-based ensemble and traditional machine learning classifiers across all cohorts. The comprehensive comparative analysis validates our choice of Extra Trees as the optimal algorithm for the proposed framework.

The quantitative comparison of proposed model with other tree-based ensemble classifiers was carried out and results are reported in **Table S3**. This comparative analysis demonstrates that Extra Trees (ET) consistently outperforms the other tree-based algorithms across most metrics and datasets. The quantitative comparative evaluation of proposed model with traditional machine learning (ML) classifier is reported in **Table S4**. This analysis reveals that none of the traditional ML classifiers were able to effectively capture the underlying patterns, as evidenced by their lower performance across all metrics compared to Extra Trees across all three cohorts, highlighting the limitations of traditional models for our cohort. It is important to note that all classifiers were hyperparameter-tuned using Grid Search with a 10-fold stratified cross-validation to ensure a fair and robust evaluation of their capabilities.

**Table S1. CT Scanners and Parameters of 4 Centers**

| Parameters | Center A | Center B | Center C | Center D |
| --- | --- | --- | --- | --- |
| CT scanners | Toshiba Aquilion | GE LightSpeed VCT | Siemens  Somatom drive | Canon Aquilion CXL |
|  | GE Discovery CT750 HD | Siemens Somatom Definition | Siemens Somatom Force | Siemens Somatom Force |
|  | Philips iCT 256 | Toshiba Aquilion Prime | Siemens Biograph mCT |  |
|  | Siemens Somatom Force |  |  |  |
| Tube current (mA) | 200-250 | 120-200 | 140-210 | 70-300 |
| Tube voltage (kV) | 120 | 120 | 120 | 80-130 |
| Slice Thickness (mm) | 1.0 | 1.25 | 1.0 | 1.0 |
| Pixel spacing | 0.578-1.083 | 0.527-0.793 | 0.683-0.839 | 0.563-0.683 |

**Table S2. Quantitative Comparison with ET**

| **Classifier** | **Internal Validation AUC (95% CI)** | **Centre D (95% CI)** | **Centre E (95% CI)** | **De-long’s p-value (when compared with ET )** |
| --- | --- | --- | --- | --- |
| Random Forest | 0.8935 (0.8169–0.9565) | 0.8780 (0.7537–0.9839) | 0.8074 (0.6175–0.9576) | 2.88 × 10⁻³ |
| Bagging | 0.8867 (0.8137–0.9488) | 0.8976 (0.7941–0.9905) | 0.7880 (0.6088–0.9447) | 1.49 × 10⁻⁴ |
| Gradient Boosting | 0.8405 (0.7583–0.9122) | 0.8736 (0.7364–0.9839) | 0.8444 (0.7044–0.9606) | <1 × 10^⁻25^ |
| XGBoost | 0.8559 (0.7738–0.9280) | 0.8519 (0.7291–0.9598) | 0.7815 (0.6214–0.9211) | 3.97 × 10⁻¹³ |
| LR | 0.6287 (0.5157–0.7409) | 0.5643 (0.3727–0.7522) | 0.6204 (0.4589–0.7803) | < 1 × 10^⁻25^ |
| SVM | 0.6147 (0.4909–0.7260) | 0.5229 (0.3333–0.7166) | 0.6222 (0.4431–0.7914) | < 1 × 10^⁻25^ |
| KNN | 0.7545 (0.6643–0.8406) | 0.6057 (0.4505–0.7609) | 0.6019 (0.4177–0.7714) | < 1 × 10^⁻25^ |
| GNB | 0.5237 (0.4027–0.6438) | 0.5490 (0.3714–0.7357) | 0.5333 (0.3385–0.7364) | < 1 × 10^⁻25^ |
| LDA | 0.6258 (0.5145–0.7392) | 0.5621 (0.3698–0.7548) | 0.6259 (0.4683–0.7877) | < 1 × 10^⁻25^ |
| ET | 0.9168 (0.8510–0.9704) | 0.8769 (0.7478–0.9835) | 0.8454 (0.7018–0.9680) | N/A |

**Table S3. Quantitative comparison amongst various tree-based ensemble methods**

| Classifier | Dataset | AUC-ROC | Accuracy | Sensitivity | Specificity | F1-Score | Precision |
| --- | --- | --- | --- | --- | --- | --- | --- |
| Random Forest | Internal Validation | 0.894 | 0.851 | 0.742 | 0.889 | 0.712 | 0.697 |
|  | Centre D | 0.878 | 0.864 | 0.765 | 0.926 | 0.813 | 0.867 |
|  | Centre E | 0.807 | 0.882 | 0.733 | 0.944 | 0.786 | 0.846 |
| Bagging | Internal Validation | 0.887 | 0.851 | 0.742 | 0.889 | 0.719 | 0.697 |
|  | Centre D | 0.898 | 0.886 | 0.765 | 0.963 | 0.839 | 0.929 |
|  | Centre E | 0.788 | 0.882 | 0.733 | 0.944 | 0.786 | 0.846 |
| Gradient Boosting | Internal Validation | 0.843 | 0.851 | 0.581 | 0.944 | 0.667 | 0.783 |
|  | Centre D | 0.869 | 0.886 | 0.706 | 1.000 | 0.828 | 1.000 |
|  | Centre E | 0.844 | 0.882 | 0.733 | 0.944 | 0.786 | 0.846 |
| *XGBoost | Internal Validation | 0.872 | 0.744 | 0.839 | 0.711 | 0.627 | 0.500 |
|  | Centre D | 0.867 | 0.796 | 0.882 | 0.741 | 0.769 | 0.682 |
|  | Centre E | 0.817 | 0.843 | 0.733 | 0.889 | 0.733 | 0.733 |
| *Extra Trees | Internal Validation | 0.917 | 0.917 | 0.839 | 0.944 | 0.839 | 0.839 |
|  | Centre D | 0.877 | 0.864 | 0.765 | 0.926 | 0.813 | 0.867 |
|  | Centre E | 0.845 | 0.824 | 0.733 | 0.861 | 0.71 | 0.688 |

*XGBoost- Extreme Gradient Boosting

*Extra Tress- Extremely Randomized Trees

**Table S4. Quantitative comparison of Extra Tree Classifier with Traditional ML Classifiers**

| Classifier | Dataset | AUC-ROC | Accuracy | Sensitivity | Specificity | F1-Score | Precision |
| --- | --- | --- | --- | --- | --- | --- | --- |
| *LR | Internal Validation | 0.6287 | 0.6860 | 0.2903 | 0.8222 | 0.3214 | 0.3600 |
|  | Centre D | 0.5686 | 0.6818 | 0.3529 | 0.8889 | 0.4615 | 0.6667 |
|  | Centre E | 0.6204 | 0.5686 | 0.8667 | 0.4444 | 0.5417 | 0.3939 |
| *LDA | Internal Validation | 0.6258 | 0.6860 | 0.2903 | 0.8222 | 0.3214 | 0.3600 |
|  | Centre D | 0.5621 | 0.6818 | 0.3529 | 0.8889 | 0.4615 | 0.6667 |
|  | Centre E | 0.6259 | 0.5686 | 0.8667 | 0.4444 | 0.5417 | 0.3939 |
| *KNN | Internal Validation | 0.7545 | 0.6033 | 0.8387 | 0.5222 | 0.5200 | 0.3768 |
|  | Centre D | 0.6057 | 0.5682 | 0.6471 | 0.5185 | 0.5366 | 0.4583 |
|  | Centre E | 0.6019 | 0.6863 | 0.5333 | 0.7500 | 0.5000 | 0.4706 |
| *GNB | Internal Validation | 0.5237 | 0.4050 | 0.5484 | 0.3556 | 0.3208 | 0.2267 |
|  | Centre D | 0.5490 | 0.5455 | 0.7647 | 0.4074 | 0.5652 | 0.4483 |
|  | Centre E | 0.5333 | 0.6471 | 0.5333 | 0.6944 | 0.4706 | 0.4211 |
| *SVM | Internal Validation | 0.6147 | 0.6942 | 0.3548 | 0.8111 | 0.3729 | 0.3929 |
|  | Centre D | 0.5229 | 0.6364 | 0.4118 | 0.7778 | 0.4667 | 0.5385 |
|  | Centre E | 0.6222 | 0.7647 | 0.4467 | 0.8889 | 0.5385 | 0.6364 |
| *Extra Trees | Internal Validation | 0.917 | 0.917 | 0.839 | 0.944 | 0.839 | 0.839 |
|  | Centre D | 0.877 | 0.864 | 0.765 | 0.926 | 0.813 | 0.867 |
|  | Centre E | 0.845 | 0.824 | 0.733 | 0.861 | 0.71 | 0.688 |

*GNB- Gaussian Naïve Bayes

*SVM- Support Vector Machines

*Extra Tress- Extremely Randomized Trees

*LR- Logistic Regression

*LDA- Linear Discriminant Analysis

*KNN- K-Nearest Neighbors

1. ***The difference in SHAP values between the combined plot and individual class plots occurs because: -***

   1. ***Combined Plot: Uses all data points together, calculating SHAP values based on the global feature distributions and interactions across both classes.***
   2. ***Individual Class Plots: Calculate SHAP values separately for each class, considering only the data points within that class. The model's behavior can vary between classes due to:-***
      - ***Different feature distributions***
      - ***Varying feature interactions***
      - ***Class-specific patterns***

   [↑](#footnote-ref-1)
